# Long-Lasting Activity of ERK Kinase Depends on NFATc1 Induction and Is Involved in Cell Migration-Fusion in Murine Macrophages RAW264.7

**DOI:** 10.3390/ijms21238965

**Published:** 2020-11-25

**Authors:** Roberta Russo, Selene Mallia, Francesca Zito, Nadia Lampiasi

**Affiliations:** Istituto per la Ricerca e l’Innovazione Biomedica (IRIB), Consiglio Nazionale delle Ricerche, Via Ugo La Malfa 153, 90146 Palermo, Italy; roberta.russo@irib.cnr.it (R.R.); selenemallia93@gmail.com (S.M.); francesca.zito@irib.cnr.it (F.Z.)

**Keywords:** MAPKs, PD98059, FR180204, gene expression, siRNA, RANKL, osteoclast hallmarks

## Abstract

Macrophages are mononuclear cells that become osteoclasts (OCs) in the presence of two cytokines, macrophage colony-stimulating factor (M-CSF), and receptor activator of NF-κB ligand (RANKL). RANKL binding to its specific receptor RANK leads to OCs differentiation mainly by nuclear factor of activated T-cells cytoplasmic 1 (NFATc1). In our previous study, the analysis of the protein network in NFATc1-knockdown cells, using the Ingenuity Pathway Analysis (IPA), showed a link between NFATc1 and Mitogen-activated protein kinase kinase (MEK)-extracellular receptor kinase (ERK) signaling pathway. Therefore, this study aimed to extend our knowledge of the relationship between NFATc1 and the ERK. Here, we demonstrate that delayed ERK1/2 phosphorylation in pre-OC RANKL-induced depends on NFATc1. Indeed, the knockdown of NFATc1 reduced the phosphorylation of ERK1/2 (60%) and the pharmacological inhibition of the ERK1/2 kinase activity impairs the expression of NFATc1 without preventing its translocation into the nucleus. Furthermore, silencing of NFATc1 significantly reduced RANKL-induced migration (*p* < 0.01), and most pre-OCs are still mononuclear after 48 h (80 ± 5%), despite the presence of actin rings. On the other hand, the inhibitors FR180204 and PD98059 significantly reduced RANKL-induced cell migration (*p* < 0.01), leading to a reduction in the number of multinucleated cells. Finally, we suggest that long-lasting ERK activity depends on NFATc1 induction and is likely linked to cell migration, fusion, and OC differentiation.

## 1. Introduction

OCs are multinucleated giant cells formed from hematopoietic progenitors in the monocyte/macrophage lineage [1]. Under the influence of M-CSF, these progenitor cells survive and proliferate to expand themselves, becoming pre-OCs [2] while, under the influence of RANKL, pre-OCs migrate, fuse, and ultimately differentiate in mature OCs [3]. The cytokine RANKL is supplied by osteoblasts and/or osteocytes and binds to its receptor RANK present on pre-OCs [4]. The pre-OCs are mononuclear cells, positive for the presence of Tartrate-resistant acid phosphatase (TRAP+), that fuse to form multinucleated mature TRAP + OCs, active for bone resorption [5]. RANKL-RANK binding leads to RANK aggregates in trimers and this association transduces the intracellular signal through the recruitment of adapter proteins, including TNF receptor-associated factor 6 (TRAF6) [6,7]. TRAF6 is a crucial signaling protein that functions for different pathways, from adaptative and innate immunity to bone metabolism [8]. Many of its physiological responses are mediated by activation of nuclear factor kappa-light-chain-enhancer of activated B cells (NF-κB) and mitogen-activated protein kinases (MAPKs) signaling pathways. In osteoclastogenesis, TRAF6 stimulates a cascade of transcription factors, including activator protein-1 (AP-1), c-Fos, microphthalmia-associated transcription factor (Mitf), PU.1, nuclear factor of activated T-cells cytoplasmic 2 (NFATc2) [9,10]. It is likely that NF-κB, c-Fos/AP-1, and NFATc2 contribute to the basal expression of NFATc1 waiting for the self-amplification phase [11]. NFATc1 induction and amplification regulate mRNA levels of target genes driving fusion and function. Among all the transcription factors already present in pre-OCs, NFATc1 is the only one strongly up-regulated and necessary-sufficient to induce differentiation of pre-OCs into OCs following RANKL-stimulation [11,12].

MAP kinases are important key molecules for the transduction of external signals into internal cellular responses. Often, they promote different responses in the same cell or the same response in different cells. These kinases are mainly represented by c-Jun N-terminal Kinase (JNK1/2), p38, and extracellular receptor kinase 1/2 (ERK1/2), which are downstream activated by RANKL [3]. The transient activation of these MAP kinases finally induces the expression of various OC marker genes including NFATc1 [13,14,15]. Bone marrow macrophages (BMMs), as pre-OCs, need M-CSF and RANKL cytokines to differentiate in OCs, whereas RAW 264.7 cells need only RANKL. M-CSF mainly promotes cellular proliferation, acting through ERK1/2-activation, whereas RANKL induces a switch versus differentiation [16]. It has been reported that RANKL, but not M-CSF, also induced a delayed (24 h) activation of p38 MAPKs that coincided with the initiation of OCs differentiation [16,17]. In particular, the axis RANKL-RANK-TRAF6-p38 MAP kinase promotes the expression of osteoclastogenic transcription factors, including NFATc1 [16]. However, in our previous study, the analysis of the protein network in NFATc1-knockdown RAW 264.7 cells, using the IPA, showed a link between NFATc1 and MEK-ERK signaling pathway [17].

Therefore, in this study, we explored the above-mentioned link, by investigating the relationship between NFATc1 expression and ERK1/2 activation and suggested that NFATc1 promotes a delayed and sustained ERK1/2 phosphorylation linked to cell migration and fusion.

## 2. Results

### 2.1. ERK Activation in RAW 264.7 Cells RANKL-Stimulated

**Figure 1:**
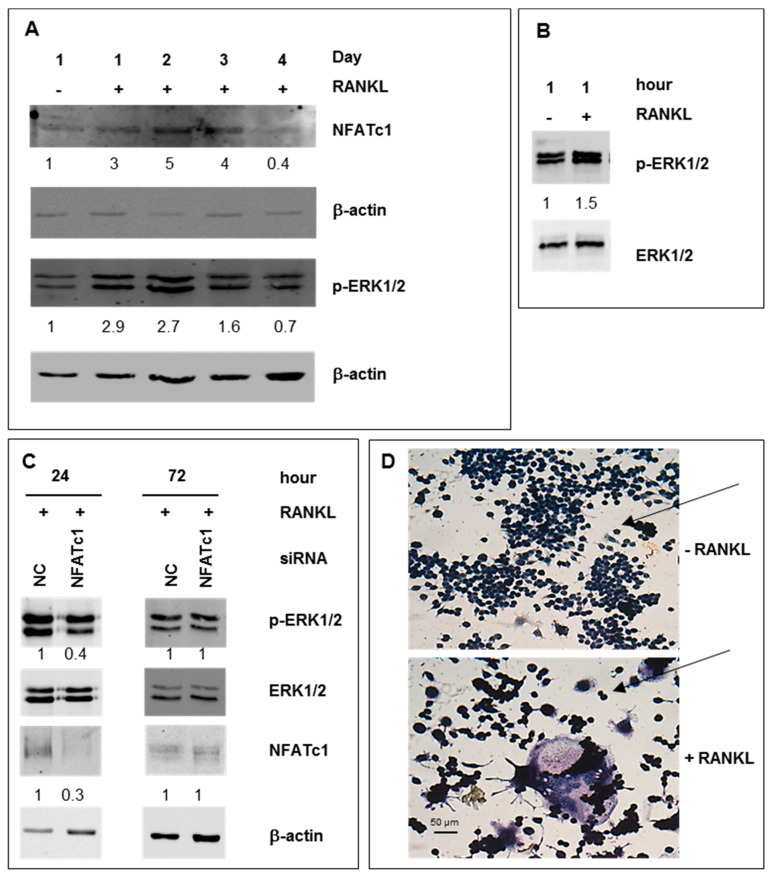
ERK1/2 phosphorylation depends on NFATc1 expression during osteoclast differentiation. RAW 264.7 macrophages were untreated or treated with RANKL for a different time and then Western blot analysis was performed for (**A**) NFATc1 and ERK1/2 phosphorylation during osteoclast differentiation (day 1–4). (**B**) ERK1/2 phosphorylation (1 h). (**C**) NFATc1 and ERK1/2 phosphorylation in cells transfected with non-correlated (NC) siRNA and NFATc1 siRNA (24–72 h). The numbers represent a fold of difference with control samples (−RANKL or NC-siRNA) arbitrarily set at 1.0. Each membrane was probed for β-actin as a loading control. The data shown represent two independent experiments with comparable outcomes. (**D**) RAW 264.7 cells were exposed or unexposed to RANKL for 4 days and then analyzed for TRAP staining. Magnification 20×. Scale bar 50 µm. The arrows indicate single cells.

We followed the differentiation of pre-OCs in mature cells during four days and evaluated NFATc1 expression and ERK1/2 phosphorylation. As shown in Figure 1A, in the presence of RANKL, NFATc1 expression significantly enhanced on the first day (3-fold), having a peak on the second-third day (5–4-fold) to decrease below the basal levels on the next day. Interestingly, the ERK1/2 activation showed a similar temporal kinetic pattern suggesting a potential relationship between them (Figure 1A). Then, we evaluated the expression profiles of ERK1/2 phosphorylation after RANKL exposure for 1 h. As expected, RANKL treatment significantly increased ERK1/2 phosphorylation (Figure 1B). To study the relationship between NFATc1 and ERK activation, we depleted RANKL-treated cells of NFATc1 mRNA by using specific siRNA and analyzed the phosphorylated forms of ERK1/2. We performed the analysis after 24 h of RANKL-induction, as it is the best time for the NFATc1-increase/ERK-phosphorylation and the beginning of pre-OCs differentiation. After 24 h, Figure 1C shows a significant decrease of the phosphorylated forms (60%), whereas the unphosphorylated form did not change, suggesting that NFATc1 induction controls late ERK activation (24 h). As a control, p-ERK1/2 in non-correlated (NC) siRNA and NFATc1-siRNA cells in basal conditions (without RANKL) are shown in Supplementary Figure S1. Conversely, as expected, the restored NFATc1 expression 72 h after transfection, due to the transient transfection, recovered ERK1/2 phosphorylation (Figure 1C). The formation of mature multinucleated OCs after 4 days of RANKL-exposition was confirmed by TRAP staining (Figure 1D).

### 2.2. RANKL-Induced Osteoclast Hallmarks Expression at 24 h

Then, we explored the expression of the main OC hallmarks RANKL-induced after 1 h and 24 h exposure. The expression of *NFATc1* mRNA is induced by RANKL at 24 h but not at 1 h (Table 1). Expression levels of *Acp5 /TRAP*, *MMP9, CtsK, DC-STAMP,* with exception of *TRAF6,* significantly increased after 24 h of exposition to RANKL, whereas after 1 h of exposition their expression did not change compared to the basal levels, except for *DC-STAMP* (Table 1).

### 2.3. Effects of ERK1/2 Inhibitors on Osteoclastogenesis

**Figure 2:**
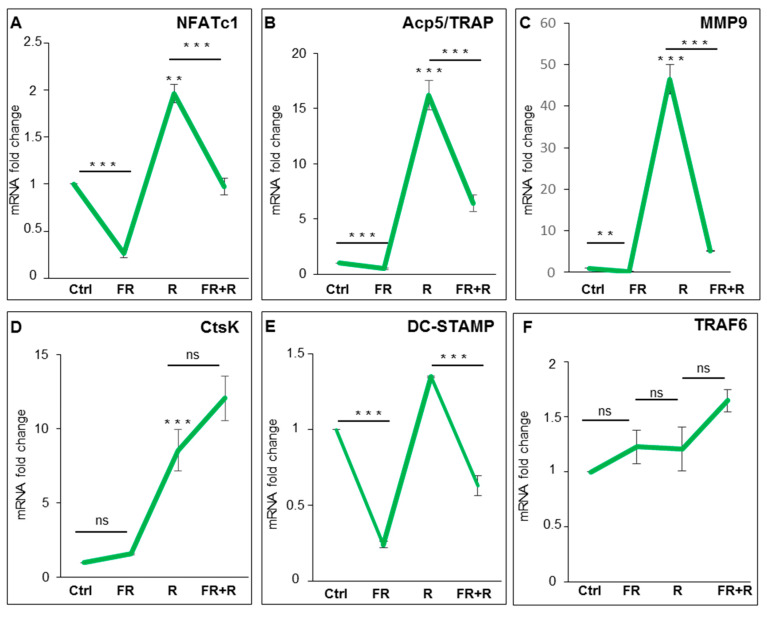
Effects of FR180204 on osteoclast hallmarks expression. Cells were untreated (Ctrl) or pretreated for 1 h with FR180204 (FR) (50 µM) and then treated with RANKL (R) (24 h). QPCR results of (**A**) *NFATc1*; (**B**) *Acp*5/TRAP; (**C**) *MMP9*; (**D**) *CtsK*; (**E**) *DC-STAMP*; (**F**) *TRAF6*. The mRNAs expression is presented as relative values of treated cells with respect to those of control cells. GAPDH was used as a housekeeping gene. The results shown are the means ± SD of three experiments (each of which was performed in triplicate). ** *p* < 0.01 and *** *p* < 0.001 each agent alone versus control (Ctrl), association (FR + R) versus each agent alone; ns—not significant.

**Figure 3:**
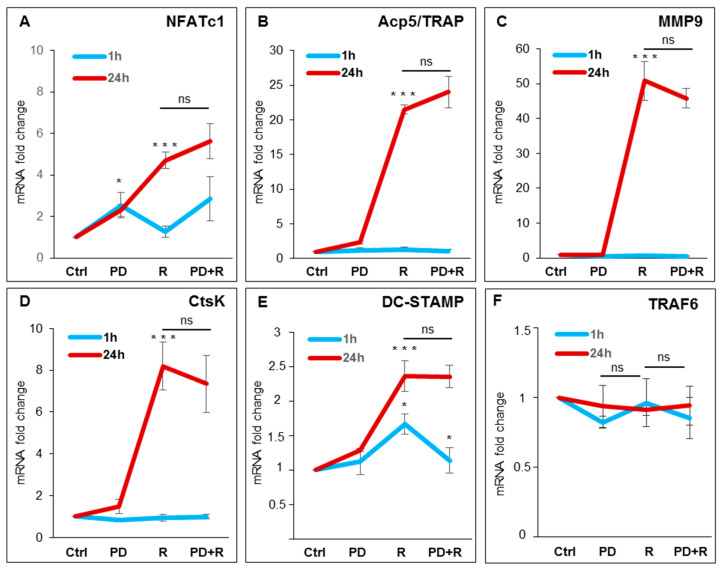
Effects of PD98059 on osteoclast hallmarks expression. Cells were untreated or pretreated for 1 h with PD98059 (PD) (50 µM) and then treated with RANKL (R) for 1 h and 24 h. QPCR results of (**A**) *NFATc1;* (**B**) *Acp*5/TRAP; (**C**) *MMP9*; (**D**) *CtsK;* (**E**) *DC-STAMP;* (**F**) *TRAF6.* The mRNAs expression is presented as relative values of treated cells with respect to those of control cells. GAPDH was used as a housekeeping gene. The results shown are the means ± SD of three experiments (each of which was performed in triplicate). * *p* < 0.05 and *** *p* < 0.001 each agent alone versus control (Ctrl), association (PD + R) versus each agent alone; ns—not significant.

To explore the role of ERK1/2 activation on OC differentiation, we used FR180204 (FR), a potent and selective adenosine triphosphate (ATP) inhibitor of ERK1 and ERK2, which inhibits the activity of ERK1 and ERK2 kinases [18]. ERK1/2 phosphorylation is not affected by treatment with FR180204 (10–50 µM) after induction with RANKL (Figure S2), as already reported by other authors in other model systems [18,19]. We performed qPCR analyses on cells pre-treated with 50 µM FR180204 for 1 h and then with RANKL for 24 h in the presence of the inhibitor. Figure 2A–F shows a strong significant reduction in the expression levels of *NFATc1*, *Acp5/TRAP*, *MMP9*, and *DC-STAMP,* with the exception of both *CtsK* and *TRAF6*, compared to the treatment with the cytokine alone. To further analyze the role of ERK phosphorylation in OC differentiation, we performed a qPCR analysis of RANKL-induced cells in the presence of a specific MEK-ERK1/2 pharmacological inhibitor (PD98059). First, we used three different concentrations of PD98059 (10, 30, and 50 µM), to identify the dose needed to inhibit ERK1/2 phosphorylation after 1 h and 24 h, as compared to control cells and to the total ERK1/2 levels that did not change during the treatments. In the following, we will use 50 µM PD98059, which partially (40% reduction) but significantly reduced ERK1/2 phosphorylation (Figure S2). Then, we examined the effects of PD98059 on osteoclastogenic hallmarks. As a result, pretreatment with PD98059 reduced neither *NFATc1* expression nor the OC hallmarks after 24 h of exposure with RANKL (Figure 3A–F), except the expression of *DC-STAMP* after 1 h of exposure with RANKL compared to treatment with the cytokine alone (Figure 3E).

### 2.4. Effects of ERK1/2 Inhibitors on NFATc1 Nuclear Translocation

**Figure 4:**
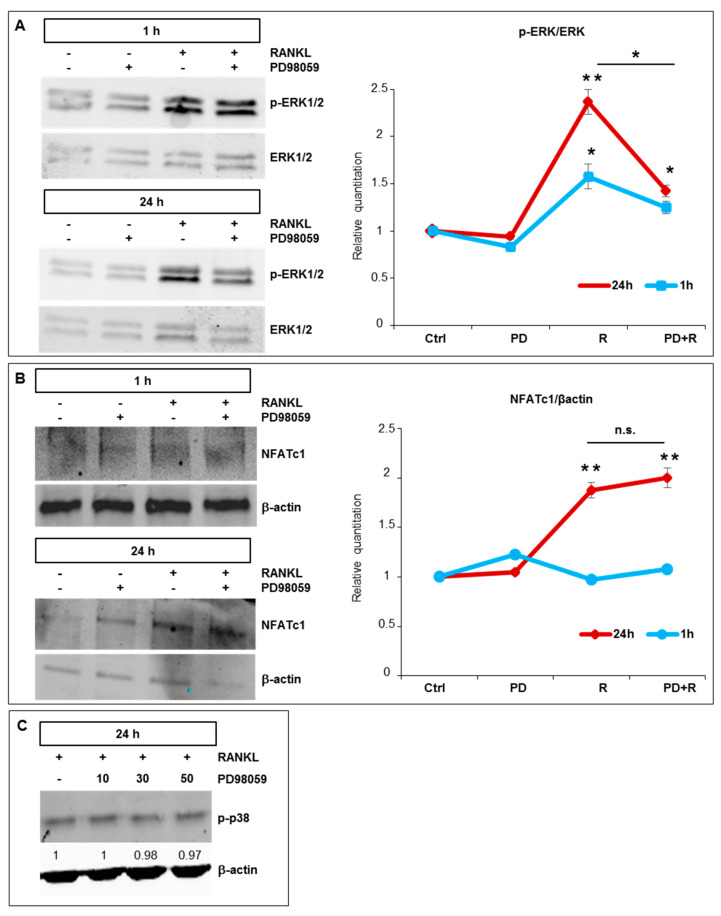
PD98059 does not affect NFATc1 expression RANKL-induced. Cells were exposed to RANKL (1 h and 24 h) (R) in the presence/absence of PD98059 (50 µM) (PD) for 1 h and then (**A**) p-ERK1/2 and ERK1/2, (**B**) NFATc1 proteins were analyzed. β-actin was a loading control. The data shown represent two independent experiments with comparable outcomes. * *p* < 0.05, ** *p* < 0.01 each agent alone versus control, association (PD + R) versus each agent alone; ns—not significant. (**C**) Western blot analysis of the p-p38 MAP kinase protein in cells pre-treated for 1 h with PD98059 (10, 30 and 50 µM) and then treated with RANKL (50 ng/mL) for 24 h. β-actin was a loading control. The numbers represent a fold of difference with RANKL arbitrarily set at 1.0. The data shown represent two independent experiments with comparable outcomes.

To further clarify the action mechanism of PD98059 on NFATc1 expression, we performed a Western blot of proteins extracted from cells treated with the inhibitor, with or without RANKL, for 1 h and 24 h. As expected, RANKL induced ERK1/2 phosphorylation after 1 h and 24 h of exposure, while PD98059 partially but significantly reduced RANKL-induced ERK phosphorylation after 24 h (Figure 4A). RANKL treatment-induced NFATc1 protein expression at 24 h, while there is no detectable increase after 1 h compared to the basal levels (Figure 4B). Furthermore, the association between PD98059 and RANKL did not reduce the expression levels of NFATc1 protein at any analyzed times compared to RANKL treatment alone (Figure 4B). To confirm the specificity of the PD98059 action on ERK1/2 phosphorylation, we analyzed its potential effects on the phosphorylation of p38 MAP kinase and, as expected, found that MEK-ERK1/2 inhibitor did not affect it, as compared with control cells (+RANKL/−PD98059; Figure 4C).

**Figure 5:**
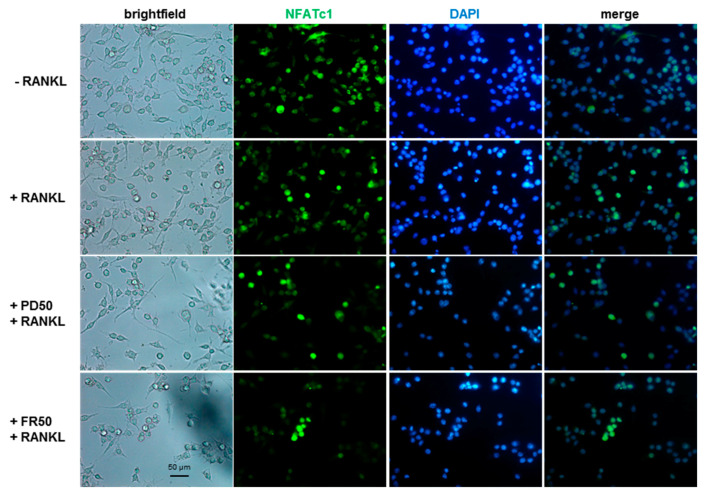
PD98059 and FR180204 do not impair NFATc1 nuclear localization RANKL-induced. Cells were untreated or pretreated with PD98059 or FR180204 (50 µM) for 1 h and then cultured with RANKL (50 ng/mL) for 24 h. Immunofluorescence analysis of NFATc1 subcellular localization without (−RANKL) or with RANKL (+RANKL) are presented. The data shown represent two independent experiments with comparable outcomes. Magnification 40×. Scale bar 50 µm.

To analyze the effects of both inhibitors on NFATc1 nuclear translocation, we performed immunofluorescence (IF) experiments in cells pretreated with either FR180204 or PD98059 (1 h) and subsequently treated with RANKL for 24 h. As shown in Figure 5, RANKL treatment alone (+RANKL) induced NFATc1 translocation in about 6.7% of the cell’s population, with respect to untreated cells (−RANKL), and the presence of the inhibitors does not significantly affect the nuclear translocation of NFATc1 induced by RANKL, which occurs regularly.

### 2.5. Effects of NFATc1 Depletion on Osteoclastogenesis

**Figure 6:**
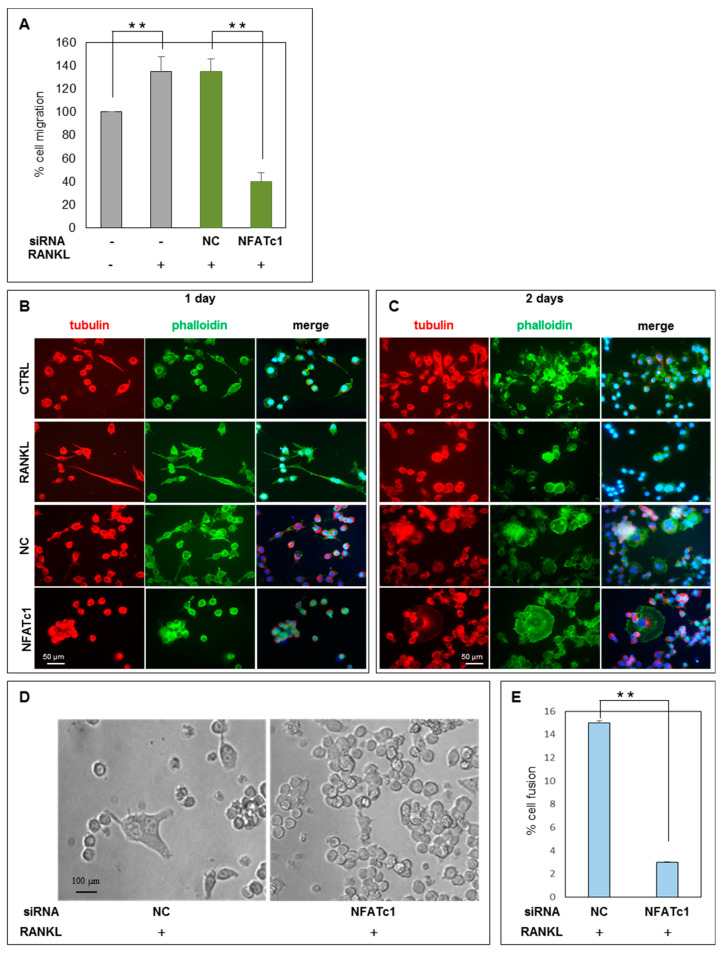
NFATc1 affects cell migration and fusion. RAW 264.7 cells were transfected with non-correlated (NC) siRNA and NFATc1 siRNA and then RANKL-induced (24 h). (**A**) The effects of NFATc1 siRNA on cell migration were analyzed by trans-well assay. Data are expressed as the percentage of migrated cells and are the mean ± SD of two separate experiments (each of which was performed in duplicate). ** *p* < 0.01 versus control or NC siRNA. Cells were untreated or treated with RANKL (50 ng/mL) and were transfected or not with NC or NFATc1 siRNAs. (**B**) Day 1 after RANKL addition; (**C**) Day 2 after RANKL addition; RAW 264.7 cells were fixed and stained with phalloidin and anti-tubulin antibody. The number of cells with filopodia were counted in different fields. Data are expressed as a percentage of filopodia-cells/cells and are the mean + SD of two separate experiments with comparable outcomes. Magnification 20×. Scale bar 50 µm. (**D**) The effects of NFATc1 siRNA on cell fusion after 24 h RANKL treatment were analyzed by microscope and cells were observed in different fields. Magnification 40×. Scale bar 100 µm. (**E**) The number of fused cells that have >2 nuclei were counted in different fields. Data are expressed as the percentage of fused cells and are the mean ± SD of three separate experiments (each of which was performed in duplicate). ** *p* < 0.01 versus control cells.

RAW 264.7 cells RANKL-treated and NFATc1-depleted showed impaired transcription of many NFATc1 target genes [17]. Among them, some genes coding for proteins linked to cell migration as well as cell-cell fusion. Thus, we evaluated the effects of NFATc1 knockdown on RAW 264.7 cells migration using a trans-well assay. Untransfected cells treated with RANKL for 24 h showed a significant increase in the number of migrated cells compared to non-stimulated control cells (ctrl, −RANKL) (Figure 6A). A similar increase was observed in cells transfected with NC siRNA after 24 h RANKL treatment. On the contrary, cells transfected with NFATc1 siRNA significantly reduced RANKL-induced migration by 70% (*p* < 0.01) (Figure 6A). The effects of NFATc1 silencing on the early steps of cell-cell fusion were evaluated by microscope analysis. We stained NFATc1-silenced cells treated with RANKL for 1 and 2 days with Alexa Fluor-488 phalloidin (green) and anti-tubulin antibody (red). We found that most of the mononuclear RANKL-stimulated cells (about 68% ± 2) showed filopodia on the 1st day, compared to control (CTRL) (about 31% ± 6) (Figure 6B). Similarly, cells transfected with NC siRNA and treated with RANKL for 1D showed the presence of filopodia (about 70% ± 5) (Figure 6B). On the contrary, the majority part of the cells transfected with NFATc1 siRNA (1D) appeared to be round-shaped and without filopodia (about 80% ± 5). After 2 days of RANKL treatment, numerous untransfected cells, as well as NC siRNA-transfected cells, contained 2–3 nuclei and possessed podosomes assembled into small actin rings (Figure 6C). Interestingly, all the NFATc1 siRNA-transfected cells were mononuclear and some seemed to adhere to each other, while spread out and formed actin rings after 2 days of RANKL treatment but none of them seemed to be fused (Figure 6C). We counted the number of multinucleated cells in different fields after 2 days of RANKL-treatment by microscope. Figure 6D shows representative fields of cells transfected with NC or NFATc1 siRNAs, respectively. Within the NC siRNA cell population, numerous cells with 2–3 nuclei were observed (equal to 15%), while in the population of NFATc1-siRNA-transfected cells the number of multinucleated cells was about 3%. Altogether, these results indicated that NFATc1 promotes cell migration and cell-cell fusion of pre-OCs.

### 2.6. Functional Analysis of ERK Activation on Cell Migration and Cell-Cell Fusion

**Figure 7:**
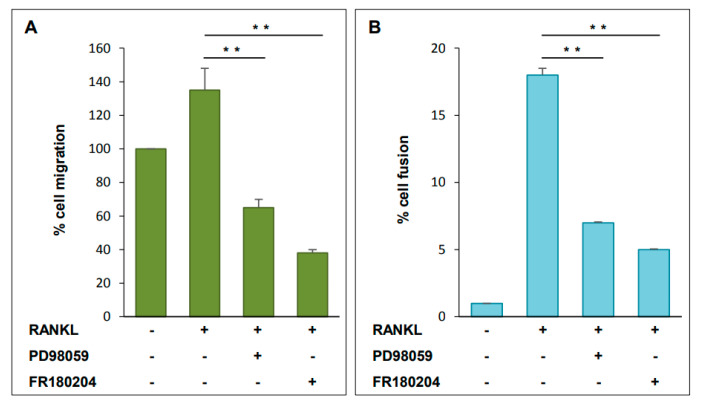
Phospho-ERK affects cell migration and fusion. RAW 264.7 cells were pretreated with PD98059 or FR180204 (50 µM) for 1 h and then RANKL was added for 24 h. (**A**) The effects on cell migration were analyzed by trans-well assay. Data are expressed as the percentage of migrated cells and are the mean ± SD of two separate experiments (each of which was performed in duplicate). ** *p* < 0.01 versus RANKL treated cells. (**B**) The effects on cell fusion were analyzed by microscope and cells were observed in different fields. The number of fused cells that have > 2 nuclei were counted in different fields. Data are expressed as the percentage of fused cells and are the mean ± SD of three separate experiments (each of which was performed in duplicate). ** *p* < 0.01 versus RANKL treated cells.

Activation of MAPK signaling results in increased phosphorylation and activation of ERK1/2, which in turn, phosphorylates several proteins associated with cell motility. To investigate the functional role of ERK1/2 during osteoclastogenesis, we used both inhibitors PD98059 and FR180204 and analyzed their effects on cell migration and cell-cell fusion. Cells were pre-treated with PD98059 or FR180204 (50 µM) for 1 h and then, in their presence, were left to migrate for 24 h with the addition of RANKL. Both inhibitors significantly reduced RANKL-induced cell migration, although PD98059 reduced it by 50% (*p <* 0.01), compared to control (RANKL-induced), while FR180204 almost completely abolished it (Figure 7A). These results suggested that NFATc1 is linked to migration likely through ERK1/2 activation.

Cell–cell fusion was analyzed on RAW 264.7 cells pretreated with PD98059 or FR180204 for 1 h and then grown in RANKL for 24 h. Multinucleated cells were visualized by the microscope and counted as described in the Material and Methods section. Both ERK1/2 inhibitors significantly reduced the number of multinucleated OCs compared to control (RANKL-induced) (Figure 7B). These results suggested that NFATc1 promotes cell fusion likely through ERK1/2 activation.

## 3. Discussion

To our knowledge, this is the first study that demonstrates a direct role of NFATc1 in phosphorylation of ERK1/2 MAP kinase; we have observed a second wave of delayed (24 to 48 h) and sustained activation of ERK1/2 consequently to NFATc1 induction. We found that ERK1/2 was activated in RANKL-stimulated RAW 264.7 macrophages and noticed that this phosphorylation is regulated during osteoclastogenesis.

OC precursors undergo cellular proliferation, through the M-CSF-Macrophage Colony Stimulating Factor I Receptor (cFMS) signaling, and cellular differentiation through the RANKL-RANK signaling [20,21]. The activation of RANKL-RANK signaling leads to many cascades mediated by JNK1/2, p38, and ERK1/2 MAP kinases [13]. For example, it has been reported that early activation of the p38 MAP kinase results in RANKL-induced OCs differentiation [13]. However, the activation of different MAP kinases during OCs maturation could be different in extent and duration. Indeed, Lee and colleagues reported that RANKL induced an early (5 to 20 min) and a delayed (8 to 24 h) activation of p38 and ERK1/2 kinases in BMMs positively related with the onset of OCs differentiation [16]. In unstimulated cells, ERK1/2 is mainly bound to MEK and is found in the cytoplasm [22,23]. The activation of MEK induces the phosphorylation of ERK1/2 on the TEY motif (Thr and Tyr residues). This dual-phosphorylation on the TEY motif leads to active site closure, alignment of key catalytic residues that interact with ATP, and remodeling of the activation loop [24]. In addition, the phosphorylation of the TEY motif, and not ERK catalytic activity, induces its nuclear translocation [22,25,26]. Indeed, a previous study has shown that the nuclear localization of ERK1/2 was reduced by the MEK-ERK PD184352 inhibitor and by mutations that prevented TEY phosphorylation [27]. In addition, ERK1/2 has other specific domains, which can be phosphorylated, capable of interacting with partner proteins, including nuclear transport proteins and cytoplasmic proteins [25,26,28,29]. In the nucleus, ERK1/2 catalyzes the phosphorylation of hundreds of transcription factors by activating or repressing them, including Erythroblast Transformation Specific (Ets), ETS Like-1 protein (Elk1), and c-Fos, while in the cytoplasm ERK1/2 can phosphorylate proteins that regulate cellular homeostasis, transport, motility. In particular, a motif distal to the catalytic site, known as D (docking)-domain (rich sequences of Lys and Arg) [30], and a docking site, called DEF (docking site for ERK, F/YXF/YP (DBP), binding pocket adjacent to the ERK catalytic site [28,31], have been described. These docking domains confer specificity in recognition and are involved in interactions with partner proteins, for example, upstream activators, substrates, scaffolds, and phosphatases. Previous studies have implicated for example the interactions of the DEF domain in ERK basal nuclear trafficking through nucleoporins [32,33], while D-domain is implicated in the interaction with transcription factors, substrates, regulators (MAP kinase kinases, MAP kinase phosphatases), and scaffolding proteins [34,35,36]. Finally, the activities of MAPK pathways are finely regulated through the de-phosphorylation of the ERK-TEY motif by phosphatases, such as double specificity phosphatases (DUSP), and through the direct binding of Raf (Raf-MEK-ERK) with the proteins from the Sprouty family (Spry) [37,38]. As already reported in the literature, DUSP and Sprouty are targeted by miRNAs [39], and miRNAs expression is known to vary during OC differentiation.

Here, we found that ERK activation followed a similar temporal expression profile of NFATc1 protein during the four days’ time of OCs differentiation in response to RANKL. Conversely, temporal patterns of expression in response to RANKL of both Mitf and the adapter protein TRAF6 (RANKL-RANK-TRAF6 axis) were dissimilar to that of ERK1/2 phosphorylation. Indeed, *Mitf* expression increases after 48–72 h (data are not shown), whereas *TRAF6* does not change at 24 h. This is not surprising, since Lee and colleagues [16] demonstrated the relationship between TRAF6 and p38 MAP kinase activation, whereas RANKL-induced ERK1/2 phosphorylation is unaffected by the presence/absence of TRAF6. Moreover, it has been demonstrated that RANK possesses two different intracellular motifs that bind TRAF6 and promotes the OCs generation. Knock-in (KI) mice for these two motifs are still able to stimulate the activation of NF-ĸB, ERK1/2, p38, and JNK pathways but they cannot stimulate c-Fos or NFATc1 in the BMMs RANKL-primed [40]. In an attempt to determine the upstream/downstream targets of ERK activation, we depleted cells for NFATc1 and then evaluated ERK1/2 phosphorylation. Our data revealed a significant decrease of ERK1/2 phosphorylation after impairment of NFATc1, suggesting a causal relationship between them. To gain insight into the role of ERK1/2 activation in RANKL-induced cells, we performed pharmacological inhibition of MEK-ERK activation (by PD98059) and ERK kinase activity (by FR180204). PD98059 is a potent and selective inhibitor of MAP kinase kinases (MAPKK), MEK1, and MEK2 [41]. The inhibitor binds to the inactive form of MAPKK and prevents its activation by upstream regulators, such as c-Raf or MEK kinase. It has been reported that the extent of MEK1/2 and consequently of ERK1/2 inhibition depended on how potently c-Raf and MEK were activated by any particular signaling cascade [41]. Here, we found that RANKL-RANK signaling cascade induces early (1 h) and late (24 h) ERK1/2 phosphorylation, and PD98059 partially (40% reduction), but significantly, affects only late ERK phosphorylation, without effects on p-p38. Interestingly, after 1 h of exposure to RANKL, NFATc1 downstream signal has not yet started, as demonstrated by the fact that the expression of NFATc1 protein, as well as mRNA, did not increase with respect to the basal level. Indeed, RANKL exposure for 1 h had no effects on OC hallmarks gene expression and of course, no changes are detected by pretreatment with PD98059, in agreement with Hotokezaka [42]. The situation is quite different after 24 h of RANKL stimulation. In fact, as expected, the cells exposed to RANKL for 24 h showed a significant increase of NFATc1 expression (protein and mRNA) compared with basal levels, and consequently, OC hallmarks gene expression significantly increased. However, cells pretreated with PD98059 and then exposed to RANKL showed no significant changes in NFATc1 expression (protein and mRNA) nor in its nuclear translocation. Moreover, the inhibition of ERK1/2 phosphorylation unaffected the expression levels of OC hallmarks genes RANKL-induced. These results are in agreement with a study from Oh and colleagues [43], which showed that the selective inhibition of ERK1/2 phosphorylation by PD98059 did not affect the expression of *Acp5/TRAP, RANK,* and *CtsK*. On the other hand, our previous study showed that NFATc1-silencing inhibited the expression of OC hallmarks and, at the same time, highlighted a link between NFATc1 and MEK-ERK signaling through the analysis of the protein network with IPA [17]. To further clarify the mechanism by which ERK1/2 activation is involved in the differentiation of OCs, we used FR180204, which directly inhibits the kinase activity of ERK. FR180204 is a potent and selective ERK1/2 inhibitor, which acts as a competitive inhibitor of ATP in the catalytic site [18]. Surprisingly, the expression of all the RANKL-induced (24 h) OC hallmarks analyzed, except both *CtsK* and *TRAF6*, but including *NFATc1*, is significantly reduced in the presence of FR180204, compared to stimulation with the cytokine alone, while it did not compromise the nuclear translocation of NFATc1.

Mononuclear OCs become multinucleated through cell–cell fusion. Cells fusion is a complicated process involving cell migration, chemotaxis, cell-cell recognition, and attachment as well as changes into a fusion competent status influenced by multiple factors and pathways [44,45]. Migration is important to bring the cells into contact with each other, promoting subsequent fusion. Accumulating evidence has revealed that ERK signaling is one of the critical regulators of cell motility through direct phosphorylation of various components of the cell motility machinery [46]. Concerning the mechanism, we investigated RANKL-induced cell migration in the presence of either PD98059 or FR180204 and found that both ERK inhibitors significantly inhibited cell migration and in turn cell-cell fusion. In accordance with Oh and colleagues, who reported that pretreatment with PD98059 completely suppressed BMMs fusion and thus the presence of multinucleated cells, even if the number of TRAP+ mononuclear cells was increased [43]. Kim and colleagues [47] showed that the early inactivation of both ERK and p38 MAP kinases reduced the expression of *Atp6v0d2d* and *DC-STAMP* and consequently cell-cell fusion in BMMs. In agreement, our results indicated that *DC-STAMP* expression, which is induced early after RANKL addition, was inhibited by PD98059 and FR180204 pretreatments. Furthermore, pretreatment with FR180204 also significantly inhibited the expression of *MMP9*, a well-known matrix metalloproteinase, usually involved in the degradation of ECM in numerous physiological processes, including, among others, cell migration [48]. In particular, MMP9 has been shown to play an important role in the recruitment of OCs [49]. Our unpublished results demonstrated a constant and progressive increase in *MMP9* expression during RANKL-induced temporal differentiation of OCs (1–4 days). Here, FR180204 pretreatment significantly reduced RANKL-induced MMP9 expression and these results are consistent with the impairment of cell migration assessed in the FR180204 trans-wells assay. In an attempt to confirm the upstream target of ERK activation, we depleted cells for NFATc1 and then we investigate cell migration and fusion. We demonstrated that NFATc1-depleted cells showed a significant reduction of cell migration as well as cell–cell fusion. In accordance with our results, Moon et al. [50].

] showed that it is precisely the impairment of NFATc1 that blocked the migration and cell-cell fusion of pre-OCs in BMM. Mature OCs acquire the bone resorption ability through a sealing zone with a filamentous actin ring [51]. Therefore, we decided to investigate the effects of NFATc1-impairment on actin ring formation (24–48 h). We found that NFATc1-depleted cells remained mononuclear and had a defective but present actin ring, showing morphological differences compared to untransfected cells or RANKL-stimulated cells.

**Figure 8:**
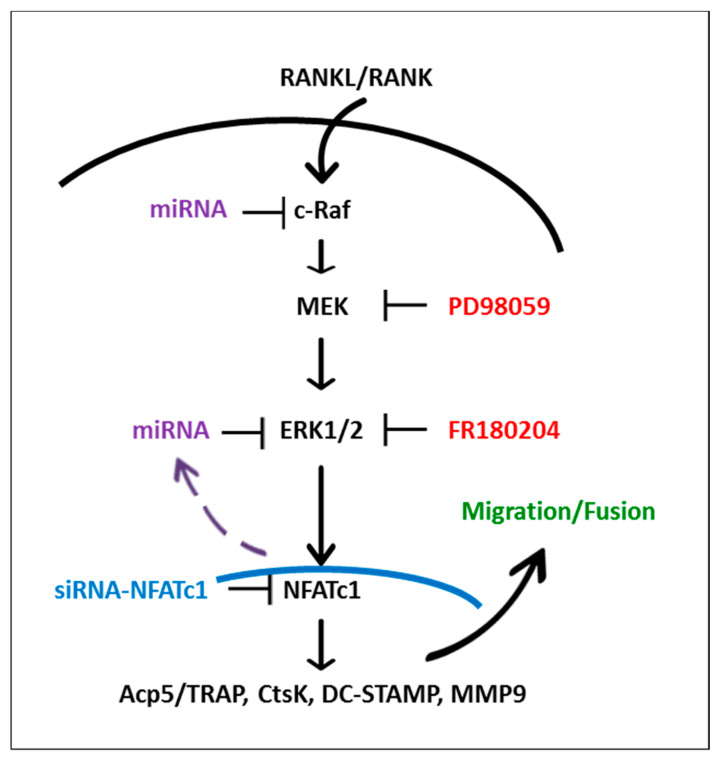
Diagram of the hypothetical mechanism controlling osteoclast differentiation involving ERK phosphorylation and NFATc1 expression. Solid arrows, known interactions (from literature or present results); dashed arrow, speculative interactions; 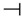, inhibition.

The apparent discrepancy in the behavior of PD98059 and FR180204 on the expression of RANKL-induced OC-specific markers, including NFATc1, with high probability, lies in the different mechanism and degree of blockage of ERK1/2 activity. PD98059 may only inhibit ERK1/2 phosphorylation, which depends on MEK-activation. Indeed, the inhibition of ERK1/2 phosphorylation using PD98059 is about 40%, and therefore there is a residual activity (60%) that could contribute to the OC hallmarks expression, including NFATc1 (Figure 8). The consequence is no effects on the expression of NFATc1 and RANKL-induced OC hallmarks, but only inhibition of cell migration and fusion, probably due to the lack of phosphorylation of the cytoplasmic partner proteins involved in these events, just as an example myosin light chain kinase (MCLK) and focal adhesion kinase (FAK), calpain, paxillin, dynamin [52,53]. On the contrary, FR180204, which completely blocks the kinase activity of ERK1/2, both that deriving from RANKL-RANK signaling cascade activation and that from NFATc1 feedback loop (NFTAc1-silencing impairs ERK phosphorylation, Figure 1C and Figure 8), may inhibit all the events downstream ERK phosphorylation, i.e., those resulting from its nuclear translocation and from the phosphorylation of cytoplasmic partner proteins. The consequence is the inhibition of the RANKL-induced OC hallmarks expression, including NFATc1, and the impairment of cell migration /fusion as shown by our results. Finally, we must take into account that the behavior of FR180204 on OCs is still unknown, since the inhibitor is relatively new and has never been used before to study OC differentiation.

Collectively, these data indicate that ERK1/2 positively regulates OCs differentiation and suggest that early (1 h) and late (24 h) ERK phosphorylation /activation may depend on different regulators. Furthermore, our results suggest a role for NFATc1-ERK activation in cell migration and the formation of multinucleated OCs. Therefore, this pathway could be a potential therapeutic target for the management of some bone diseases, such as osteoporosis and /or osteopetrosis.

## 4. Materials and Methods

### 4.1. Cell Culture and Reagents

The murine RAW 264.7 macrophage cell line was purchased from the American type culture collection (Manassas, VA, USA). Cells were grown in Dulbecco modified Eagle’s medium (DMEM, Gibco, NY, USA) with 10% heat-inactivated fetal bovine serum (FBS, Sigma-Aldrich, St. Louis, MO, USA), 100 U/mL penicillin, and 100 µg/mL streptomycin. To induce OCs differentiation, cells were suspended in an alpha-minimal essential medium (α-MEM Gibco, NY, USA) with 10% FBS (Sigma-Aldrich, St. Louis, MO, USA), 100 U/mL penicillin, and 100 µg/mL streptomycin with RANKL 50 ng/mL (Peprotech, Rocky Hill, CT, USA)RAW 264.7 differentiation to OCs can be followed in vitro in four days, considering as day 0 the addition of the cytokine RANKL, which was replaced every two days. Multinucleated osteoclasts were identified by TRAP staining. PD98059 and FR180204 were purchased from Sigma Aldrich (St. Louis, MO, USA) and were dissolved in dimethyl sulfoxide (DMSO). Cells were pre-treated with PD98059 or FR180204 for 1 h and then, in their presence, cells were treated with RANKL (50 ng/mL) for 1 h and 24 h.

### 4.2. TRAP Staining

RAW 264.7 cells, cultured as above described with RANKL (50 ng/mL) for 4 days, were fixed with 4% paraformaldehyde in PBS for 10 min at room temperature. Then, TRAP staining was carried out in accordance with the manufacturer’s instruction (Sigma-Aldrich, St Louis, MO, USA). Under light microscopy (OLYMPUS CKX31, Olympus, Tokyo, Japan), positive multinucleated cells containing more than three nuclei were considered osteoclasts.

### 4.3. Cell Fusion

To quantify the efficiency of fusion, RAW264.7 cells were seeded 70% confluent on sterile coverslips that had previously been placed in 6-well culture plates, and cultured with or without RANKL (50 ng/mL) and untransfected or transfected with siRNA for 24 h. Fusion was determined after 24 h RANKL-induction in each condition, cells were fixed and nuclei were labeled with DAPI (Molecular Probes, Eugene, OR, USA). Images were taken on an Axioskop microscope (Carl Zeiss, Oberkochen, Germany), from at least ten randomly selected fields representing the sample population for each condition and were analyzed [54]. The fusion index (%) was calculated as the ratio of the number of OCs with two or three nuclei to the total number of OCs. Experiments were repeated a minimum of three times.

### 4.4. RNA Extraction, cDNA Synthesis, and Quantitative Polymerase Chain Reaction (qPCR)

RAW 264.7 cells were cultured (1 × 10^6^ cells/well) in a 6-well plate overnight. Cells were treated with RANKL 50 ng/mL for 1, 24, and 72 h. In the experiments with inhibitors, cells were pre-treated with the inhibitors for 1 h and then treated with RANKL for 1 h or 24 h. After this time of stimulation, the cells were detached from the wells and washed once with PBS. Total RNA was isolated using the GenElute Mammalian Total RNA Miniprep Kit (Sigma-Aldrich, St. Louis, MO, USA) and quantified by using a biophotometer (Eppendorf S.r.l., Hamburg, Germany). QPCR was performed as described in the manufacturer’s manual of StepOnePlus real-time PCR, with a Comparative Threshold Cycle Method (Applied Biosystems, Life Technologies, Carlsbad, CA, USA), using SYBR Green chemistry [55]. Total RNAs (1 µg) were reverse transcribed according to the manufacturer’s instructions (Applied Biosystems, Life Technologies, Carlsbad, CA, USA). The qPCR was run as follows: 1× cycle denaturing 95 °C for 10 min for DNA polymerase activation; 38× cycles: melting 95 °C for 15 s, annealing/extension 60 °C for 60 s. The qPCR was performed in triplicate on each cDNA sample for each gene, using primers by Qiagen: QT001676692 (NFATc1), QT00166663 (TRAF6), QT00108815 (MMP9), QT00131012 (TRAP), QT01047032 (DC-STAMP), QT02589489 (CTSK2), QT01658692 (GAPDH). The threshold cycle (*CT*) values were calculated against the housekeeping gene GAPDH. At least three distinct biological samples were examined for each gene and treatment.

### 4.5. Immunofluorescence Analysis

RAW 264.7 cells were fixed with 4% paraformaldehyde in PBS for 15 min, permeabilized with 0.5% Triton X-100 in PBS for 10 min, and blocked with 1% BSA 0.1% Triton X-100 in PBS for 30 min. Primary antibodies (α-tubulin, 1:500, Sigma-Aldrich; NFATc1, 1:20, Santa Cruz, Dallas, TX, USA) and Alexa Fluor-488 Phalloidin (1:250, Invitrogen Molecular Probes, Carlsbad, CA, USA) were incubated for 2 h at room temperature, and secondary antibodies (Alexa Fluor 594 rabbit anti-mouse, 1:500; Alexa Fluor 488 rabbit anti-mouse, 1:200, Invitrogen Molecular Probes) and DAPI were incubated for 1 h. Samples labeled with α-tubulin and Alexa Fluor-488 phalloidin were observed under Axioskop 2 Plus microscope (Zeiss, Oberkochen, Germany) equipped for epifluorescence and images recorded by a digital camera system (40×). Samples labeled with NFATc1 were observed under a Nikon Eclipse 80i microscope equipped for epifluorescence and recorded by a digital camera system (40×). The percentage of cells showing NFATc1 nuclear translocation was calculated as the ratio of nuclei labeled with NFATc1 to the total number of nuclei labeled with DAPI, counted using the ImageJ software.

### 4.6. Western Blot

RAW 264.7 cells, cultured as above described for 1 h and 1, 2, 3, and 4 days (D), with RANKL (50 ng/mL), were lysed, and total proteins were extracted using RIPA buffer (Cell Signaling Inc., Beverly, MA, USA). The protein concentration of cell lysates was determined by the Bradford method. For immunoblotting, 30 µg of protein was separated on 10% SDS–polyacrylamide gels by electrophoresis and transferred to a nitrocellulose membrane (Millipore, Temecula, CA, USA). Membranes were incubated overnight at 4 °C with the following antibodies: NFATc1 (Santa Cruz, CA, USA) at 1:1000 dilution; phospho-ERK1/2 (p-ERK1/2), total ERK1/2 and phospho-p38 at 1:1000 dilution (Cell Signaling, Inc. Beverly, MA, USA); β-actin (Sigma Aldrich, St. Louis, MO, USA) at 1:5000 dilution. The secondary antibodies, Alexa Fluor 680 goat anti-rabbit (1:2000) and Alexa Fluor 800 rabbit anti-mouse (1:5000) (Molecular Probes, Life Technologies, Carlsbad, CA, USA), were incubated for 1 h at room temperature. Proteins were visualized using an Odyssey Infrared Imaging System (LI-COR) according to the manufacturer’s instructions. Densitometry analyses were conducted using the Quantity One software (Bio-Rad, Hercules, CA, USA).

### 4.7. Small Interfering RNA (siRNA) Transfection

Cells were transfected with NFATc1 siRNA, and Non-Correlated (NC) siRNA (Qiagen, Hilden, Germany) as previously reported [17]. In brief, cells (2.5 × 10^5^) were seeded onto 6-well plates in DMEM without antibiotics; 24 h later, the transfection of siRNAs was carried out with Lipofectamine RNAiMAX (Invitrogen, Carlsbad, CA, USA). All transfections were carried out with 20 µM duplex siRNA in medium without FBS and antibiotics. After 24 h, cells were split into 6-well plates and incubated with RANKL (50 ng/mL) for 24 and 72 h to perform further analysis. After this time, mRNA analysis was carried out by qPCR, protein analysis was performed by Western blot, and cell staining was performed by immunofluorescence. Experiments were repeated three times.

### 4.8. Migration Assay

Migration assays were performed in 6.5 mm diameter trans-well plates containing 8 µm pore size filters (Corning, Glendale, AZ, USA)**.** Briefly, trans-well analysis was conducted on cells transfected with siRNA or untransfected and treated with PD98059 or FR180204. Cells transfected with NC-siRNA or NFATc1-siRNA for 24 h were used for subsequent experiments. Briefly, cells were harvested and resuspended (2.0 × 10^5^ cells/mL) in serum-free growth medium. 100 µL of cell suspension (NC siRNA and NFATc1 siRNA or untransfected cells) was added to the upper chamber, whereas 800 µL of growth medium with 10% fetal calf serum and RANKL (50 ng/mL) were added in the lower chamber. For PD98059 and FR180204 treatments, the cells were seeded in the upper chamber in the presence of PD98059 or FR180204 and 1 h later RANKL was added in the lower chamber. After 24 h of incubation at 37 °C, the trans-wells were removed, the cells were fixed with 4% paraformaldehyde, permeabilized with 100% methanol, colored with crystal violet and then the cells on the upper side were scraped off. The number of migrated cells was counted in 5 random fields under a Leica microscope (Wetzlar, Germany) at 40× magnification. The experiments were repeated three times.

### 4.9. Statistical Analysis

Data are expressed as mean ± S.D. from at least three experiments and statistical analyses were performed by Student’s *t*-test. *p* < 0.05 was considered to indicate a statistically significant difference.

## Figures and Tables

**Table 1:** Gene expression in RAW 264.7 cells RANKL-stimulated for 1 h or 24 h. Data from qPCR experiments.

Gene	1 h	±SD	24 h	±SD
NFATc1	1.3	0.6	3.6	1.6
Acp5/TRAP	1.2	0.4	18.6	3.5
MMP9	0.8	0.1	46.5	7.0
CtsK	1.0	0.3	9.0	1.9
DC-STAMP	1.7	0.3	1.9	0.5
TRAF6	0.9	0.3	1.1	0.4

The values shown derive from the combination of the data present in Figure 2; Figure 3 (R). The mRNAs expression is presented as relative values of RANKL treated cells with respect to those of control cells (−RANKL). GAPDH was used as a housekeeping gene.

## Supplementary Materials

The following are available online at https://www.mdpi.com/1422-0067/21/23/8965/s1.

## Abbreviations

Osteoclasts (OCs), macrophage colony-stimulating factor (M-CSF), receptor activator of NF-κB ligand (RANKL), Ingenuity Pathway Analysis (IPA), Tartrate-resistant acid phosphatase (TRAP), TNF receptor-associated factor 6 (TRAF6), nuclear factor kappa-light-chain-enhancer of activated B cells (NF-κB), mitogen-activated protein kinases (MAPKs), activator protein-1 (AP-1), microphthalmia-associated transcription factor (Mitf), nuclear factor of activated T-cells cytoplasmic 2 (NFATc2), nuclear factor of activated T-cells cytoplasmic 1 (NFATc1), c-Jun N-terminal Kinase (JNK1/2), extracellular receptor kinase 1/2 (ERK1/2), Mitogen-activated protein kinase kinase (MEK), Bone marrow macrophages (BMMs), acid phosphatase 5, tartrate resistant (Acp5), Matrix metallopeptidase 9 (MMP9), Cathepsin K (CtsK), dendritic cell specific transmembrane protein (DC-STAMP), immunofluorescence (IF), control (CTRL), Macrophage Colony Stimulating Factor I Receptor (cFMS), Erythroblast Transformation Specific (Ets), ETS Like-1 protein (Elk1), double specificity phosphatases (DUSP), Sprouty (Spry), MAP kinase kinases (MAPKK), (Atp6v0d2d), myosin light chain kinase (MCLK), focal adhesion kinase (FAK).
