# Supplementary material for: Long-Lasting Activity of ERK Kinase Depends on NFATc1 Induction and Is Involved in Cell Migration-Fusion in Murine Macrophages RAW264.7
Source: Int J Mol Sci. 2020 Nov 25;21(23):8965. doi: 10.3390/ijms21238965 (PMC7728313; doi:10.3390/ijms21238965)
Supplement: Supplementary file 1 [file ijms-21-08965-s001.pdf]

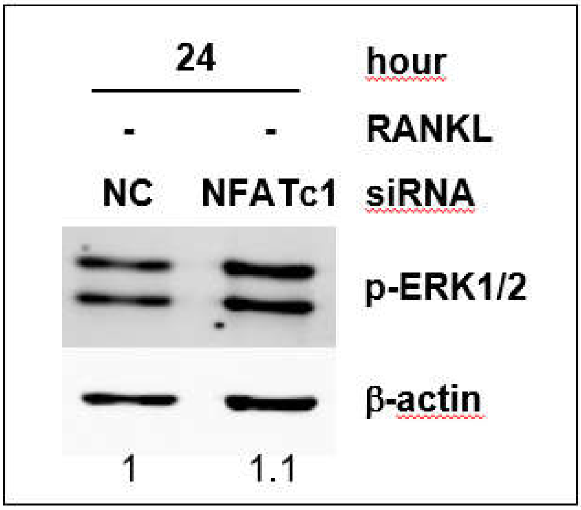

Figure S1. p-ERK in cells transfected with NC-siRNA and NFATc1-siRNA in basal conditions. Western blot of the p-ERK1/2 protein in cells transfected with NC-siRNA and NFATc1-siRNA for 24 h in the absence of RANKL. β-actin was a loading control. The numbers represent fold changes respect with NC-siRNA, arbitrarily set at 1.0. The data shown represent two independent experiments with comparable outcomes.

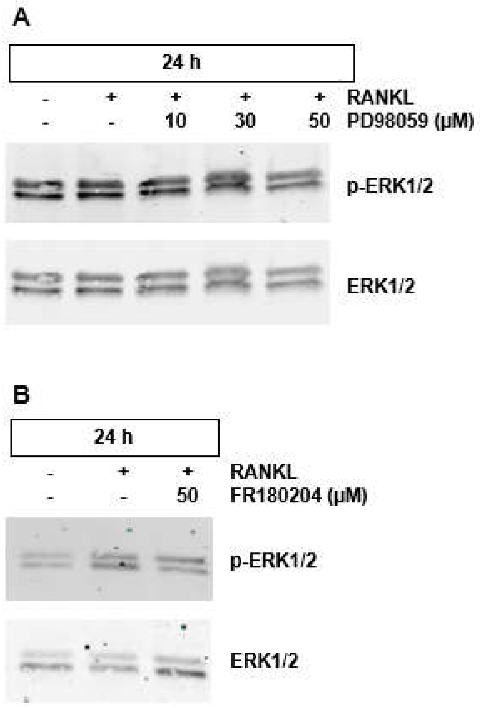

C

Sample	Fold change	± SD
-RANKL	1	0
+RANKL	2.5	0.25
+RANKL/+PD10	2.5	0.3
+RANKL/+PD30	2.3	0.15
+RANKL/+PD50	1.5	0.1
+RANKL/+FR10	2.5	0.12
+RANKL/+FR50	2.2	1.4

Figure S2. Effects of PD98059 and FR180204 on p-ERK at 24h. Western blot analysis of the p-ERK1/2 and ERK1/2 proteins in cells (**A**) pre-treated for 1 hour with PD98059 (10, 30, and 50 μM); (**B**) pre-treated for 1 h with FR180204 (50 μM) and then treated with RANKL (50 ng/ml) for 24 hours. The numbers in the table represent fold changes respect with the control (−RANKL), arbitrarily set at 1.0. The data shown represent two independent experiments with comparable outcomes.
